# Associations among personality traits, emotional states, and self-management behaviors with quality of life in type 2 diabetes: a structural equation modeling approach examining emotional mediation

**DOI:** 10.3389/fpsyg.2025.1629825

**Published:** 2025-08-29

**Authors:** Wen Fu, Jue Xu, Caixia Jiang, Shijun Liu, Cheng Yang, Xin Qiu

**Affiliations:** Department of Chronic Non-communicable Diseases Prevention and Control, Hangzhou Center for Disease Control and Prevention (Hangzhou Health Supervision Institute), Hangzhou, China

**Keywords:** diabetes mellitus, personality, emotion, self-management behaviors, quality of life, structural equation modeling

## Abstract

**Background:**

Current empirical literature demonstrates a paucity of evidence elucidating the intricate network relationships among personality traits, emotion states, self-management behaviors, and quality of life (QOL) in patients with type 2 diabetes mellitus (T2DM). This cross-sectional study aimed to investigate these relationships using structural equation modeling (SEM).

**Methods:**

A cohort of 839 T2DM patients was systematically recruited from 69 community health service centers in Hangzhou, China, between 2016 and 2020. Standardized instruments were administered to assess demographic characteristics, personality traits (Chinese Big Five Personality Inventory-15, CBF-PI-15), emotional states (Self-Rating Anxiety Scale [SAS] and Self-Rating Depression Scale [SDS]), self-management behaviors (Type 2 Diabetes Self-Care Scale, 2-DSCS), and QOL (MOS 36-Item Short-Form Health Survey, SF-36). Data analyses were performed using SPSS 26.0 and AMOS 21.0.

**Results:**

Descriptive statistics revealed the highest mean score for agreeableness (13.58 ± 2.55), whereas self-management subdomains exhibited comparatively lower scores (blood glucose monitoring: 12.17 ± 4.10; regular exercise: 12.35 ± 4.89). Significant anxiety and depressive symptoms were present in 20.4 and 28.6% of participants, respectively. Bivariate correlations showed significant positive associations between self-management behaviors and both psychological/physiological QOL dimensions, alongside negative correlations with anxiety, depression, and neuroticism. The SEM analysis yielded excellent model fit indices (*χ*^2^/df = 3.556, AGFI = 0.946, GFI = 0.967, CFI = 0.957, IFI = 0.957, RMSEA = 0.055), with anxiety emerging as the most robust predictor of QOL (*β* = −0.542), followed by depression (*β* = −0.360) and self-management behaviors (*β* = 0.342). Mediation analysis confirmed the significant intermediary roles of anxiety and depression in pathway linking self-management behaviors to QOL (indirect effects accounting for 33.70%, 30.33% of total variance).

**Conclusion:**

These findings elucidate the complex psychobehavioral mechanisms underlying QOL in T2DM patients, highlighting the critical mediating role of emotional states between self-management and QOL. The results underscore the imperative for integrated interventions targeting both emotional regulation and behavioral modification in diabetes care protocols.

## 1 Introduction

Type 2 diabetes mellitus (T2DM) currently affects approximately 500 million individuals globally (Sun et al., 2022), with projections suggesting a rise to over 600 million cases by 2040 (Basiri et al., 2023). In China, the 2020 report on Nutrition and Chronic Diseases of Chinese Residents revealed that 11.9% of Chinese adults have diabetes, underscoring a substantial public health concern (National Health Commission Disease Control and Prevention Bureau, 2021). T2DM, especially when complicated by severe chronic conditions, not only imposes a heavy economic burden on patients but also significantly deteriorates their quality of life (QOL) (Bragg et al., 2017). As a key metric in chronic disease management, QOL reflects both the physical and psychosocial consequences of T2DM. Impaired QOL may contribute to poor self-care adherence, further exacerbating glycemic dysregulation and elevating the risk of diabetes-related complications (Gaffari-Fam et al., 2020). Given these implications, healthcare systems and society must prioritize QOL assessment alongside traditional biomedical indicators to optimize comprehensive patient care (Speight et al., 2020).

Systematic reviews demonstrate that key diabetes self-management behaviors including physical activity, medication adherence, and blood glucose monitoring, significantly predict health-related quality of life (HRQL) (Teli et al., 2023). Self-management refers to the daily practices individuals adopt to control their condition and minimize its effects on physical health. Given that the onset, progression, and clinical outcomes of T2DM are influenced by psychological and behavioral factors, a multidisciplinary treatment approach is essential (Tan et al., 2020). Healthcare professionals (e.g., physicians, nurses, podiatrists, dietitians) should collaborate to develop personalized care plans, with each discipline contributing specialized expertise. Self-management strategies are recognized as vital for enhancing patient care (Ory et al., 2025). Empirical evidence supports that consistent self-management practices—such as dietary modifications, regular physical activity, glucose monitoring, and medication compliance—lead to better metabolic control. A cross-sectional study further confirmed a positive correlation between self-management behaviors and QOL, highlighting their role in enhancing overall well-being (Salzwedel et al., 2020). Thus, developing robust self-management competencies in T2DM patients represents a critical intervention strategy for optimizing health outcomes and overall well-being.

Personality traits have been shown to significantly influence self-management behaviors among individuals with T2DM (Dadras et al., 2022). As stable patterns of emotions, cognitions, and behaviors, personality serves as a crucial determinant in understanding and predicting human behavior. A Beijing-based cross-sectional study utilizing the Chinese Big Five Personality Inventory (CBF-PI-B) found significant associations among all five personality dimensions and self-management attitudes. Of particular clinical relevance, neuroticism emerged as a critical factor influencing patients’ mental health outcomes and quality of life (Zhang et al., 2019). These findings suggest that incorporating personality assessment into clinical practice could facilitate the development of personalized interventions to optimize diabetes self-management.

Personality and emotion regulation share cybernetic processing mechanisms, explaining why certain traits correlate with specific regulation strategies (Hughes et al., 2020). For instance, neuroticism predicts a greater tendency toward sadness and negative affect (Schindler and Querengässer, 2019). Anxiety and depression, recognized as prevalent mental health disorders, manifest through significant alterations in cognition, affect, and behavior that impair psychosocial daily functioning and QOL (Stevanovic et al., 2019). These psychological comorbidities not only elevate diabetes risk but also accelerate disease progression, underscoring the critical bidirectional relationship between diabetes and mental health. Epidemiological data from Chinese hospital settings indicate strikingly high prevalence rates of 51.3% for depression and 56.2% for anxiety among individuals with diabetes (Yan and Yuan, 2016). The persistent negative affect characteristic of these conditions has been shown to significantly compromise diabetes self-management capacity, resulting in deteriorated health outcomes, reduced quality of life, and increased healthcare utilization (Ozdemir and Sahin, 2020; Bayani et al., 2022). Consequently, integrated care models that address the mental health dimensions of diabetes—particularly depression and anxiety symptomatology-represent a crucial intervention approach for enhancing patient well-being and optimizing diabetes management outcomes (De Groot et al., 2016).

Previous studies have predominantly examined bivariate or trivariate relationships among personality traits, emotional states, self-management behaviors, and QOL. For example, high neuroticism is linked to maladaptive emotion regulation, worsening anxiety (Hughes et al., 2020). Self-care behaviors (particularly nutritional management) positively correlate with QOL (Babazadeh et al., 2017), while dispositional anxiety predicts poorer QOL post-diagnosis (Hall et al., 2009). One SEM analysis identified negative affectivity as adversely impacting both QOL dimensions and metabolic control in chronic disease populations (Conti et al., 2017). Notably, empirical studies examining the mediating mechanisms remain limited. A recent study utilizing 2021 PBICR data (Psychology and Behavior Investigation of Chinese Residents) revealed that personality traits exerted both direct effects on self-management and indirect effects mediated by family health and health literacy among young and middle-aged patients with chronic diseases (Lang et al., 2025). The present study proposes to address this knowledge gap by employing structural equation modeling to examine the pathways through which personality characteristics, emotional regulation, self-management behaviors influence quality of life. This analytical approach will enable a comprehensive evaluation of the collective impact of these variables on quality of life outcomes, while providing an evidence-based theoretical framework to inform targeted interventions for diabetes management optimization.

## 2 Methods

### 2.1 Research participants

From 2016 to 2020, Hangzhou conducted a community-based pilot intervention program targeting diabetes self-management. Participant recruitment was implemented through a multi-channel approach involving community mobilization, poster campaigns, and digital announcements across 69 community health centers and affiliated stations. The study employed stringent selection criteria:

Inclusion criteria: (1) Diagnosis of T2DM according to the Chinese Guidelines for the Prevention and Treatment of Type 2 Diabetes Mellitus (2020 edition) (Chinese Diabetes Society, 2021); (2) Ability to communicate effectively; (3) Voluntarily consenting to participate by signing an informed consent form.

Exclusion criteria: (1) Severe diabetes-related complications or major systemic comorbidities; (2) History of personality disorders, cognitive impairments, severe psychiatric illnesses, or substance abuse; (3) Communication disabilities.

A total of 839 eligible patients were enrolled and completed comprehensive baseline assessments before the intervention. The present study used this pre-intervention dataset for analysis. The research protocol received ethical approval from the Ethics Committee of the Hangzhou Center for Disease Prevention and Control (approval number: 2020026). All participants underwent standardized informed consent procedures, including detailed explanation of study protocols and voluntary participation agreements.

Consistent with established methodological guidelines for SEM analyses, the minimum sample size requirement was determined to be 10 times the free parameters in the hypothesized path model (Wolf et al., 2013). This study included 26 free parameters and 15 observed variables, with an initial model degree of freedom of 94. Consequently, the required sample size was a minimum of 260 participants, substantially exceeding this threshold. This ensures robust parameter estimation and model fit evaluation for our research objectives.

### 2.2 Data collection

Standardized data collection was implemented by trained general practitioners who completed a two-day training program covering the study protocol, questionnaire administration procedures, and quality control requirements. The data were collected through face-to-face interviewer-administered surveys using protocol-defined scripts. Participants received explicit instructions emphasizing the absence of correct or incorrect responses to mitigate response bias. When participants encountered comprehension difficulties, interviewers provided scripted non-directive clarification to maintain response neutrality. To ensure data quality, research staff systematically verified all completed questionnaires. Any identified omissions or inconsistencies were addressed through follow-up with participants prior to data finalization.

The comprehensive survey instrument captured multiple domains of participant characteristics, including: Sociodemographic variables (gender, age, education attainment, marital status, and the healthcare payment capacity); Psychological constructs (personality traits and emotional states); Behavior measures (diabetes self-management behaviors); Health outcomes (quality of life indicators). All assessment instruments were systematically integrated into a unified questionnaire administered in a single session. Following rigorous quality control procedures, the study obtained 839 fully completed and validated questionnaires for subsequent analysis.

### 2.3 Instruments

The Chinese Big Five Personality Inventory-15 (CBF-PI-15) is a psychometrically validated instrument adapted from short-form version of the Chinese Big Five Personality Inventory (CBF-PI-B) (Zhang et al., 2019). The 15-item scale assesses the five-factor personality model (neuroticism, conscientiousness, agreeableness, openness, and extraversion) with three items per dimension. The measure utilizes 6-point Likert-type scale (1 = “strongly disagree” to 6 = “strongly agree”), with items 2 and 5 reverse-scored to mitigate response bias. Dimension scores are computed by summing relevant items, with higher composite scores indicating greater trait manifestation. Psychometric evaluation demonstrated acceptable internal consistency across dimensions, with Cronbach’s *α* coefficients of 0.747 (neuroticism), 0.611 (conscientiousness), 0.740 (agreeableness), 0.803 (openness), and 0.738 (extraversion), collectively supporting the instrument’s reliability for research applications.

The Type 2 Diabetes Self-Care Scale (2-DSCS), originally developed by Ozdemir and Sahin (2020) and subsequently adapted by Wang Jingxuan, is a 26-item multidimensional instrument assessing six critical domains of diabetes self-management: dietary control, regular exercise, medication adherence, blood glucose monitoring, foot care, and prevention and management of hyperglycemia and hypoglycemia. Responses were recorded on a 5-point Likert scale (1 = “not at all” to 5 = “completely”). Total scores (sum of all items) range from 26 to 130, with higher scores indicate superior self-management capacity. Based on validated cut-off values, performance levels were categorized as: inadequate (<60), moderate (60–80), and optimal (>80). Psychometric analyses demonstrated excellent scale reliability, with Cronbach’s ranging from 0.82 to 0.88 across subscales, indicating strong internal consistency. Furthermore, test–retest reliability coefficients of 0.92–0.96 confirmed superior temporal stability.

The psychological assessment battery included two well-validated measures of emotional symptoms. Self-Rating Anxiety Scale (SAS) (Zung, 1971): This 20-item instrument evaluates subjective feelings of anxiety using a 4-point frequency scale (from “none or little of the time” to “most or all of the time”). Raw scores are transformed to a standardized metric (×1.25, rounded), with established clinical thresholds: Normal range (<50), Mild anxiety (50–59), Moderate anxiety (60–69), Severe anxiety (≥70), Self-Rating Depression Scale (SDS) (Zung, 1965): Similarly structured with 20 items rated on a 4-point frequency continuum (“rarely” to “continuously”), the SDS employs comparable standardization procedures with distinct clinical cutoffs: Normal range (<53), Mild depression (53–62), Moderate depression (63–71), Severe depression (≥72). For both instruments, higher standardized scores indicate greater symptom severity. Psychometric evaluations demonstrated robust internal consistency, with Cronbach’s 0.81 for the SAS and 0.82 for the SDS, confirming their reliability for clinical research applications.

The Medical Outcomes Study 36-Item Short-Form Health Survey (SF-36) (Ware and Sherbourne, 1992) is a psychometrically validated multidimensional instrument assessing eight health-related quality of life domains: (1) limitations in physical functioning, (2) limitations to usual roles due to physical problems, (3) bodily pain, (4) general health perceptions, (5) limitations in social functioning, (6) limitations to usual roles due to emotional problems, (7) vitality, and (8) general mental health. Raw scores were transformed to a 0–100 using standardized algorithms: (raw score – minimum possible score)/(possible maximum score – minimum possible score) × 100. The measure yields two composite scores: Physical Component Summary (PCS): physical functioning, role limitations due to physical problems, bodily pain, and general health perceptions; Mental Component Summary (MCS): social functioning, role limitations due to emotional problems, vitality, and general mental health. Composite scores were computed using U. S. normative factor coefficients (Ware et al., 1996), with higher scores (range: 0–100) indicating better quality of life. The Chinese version demonstrated excellent reliability (Cronbach’s *α* = 0.83), supporting its psychometric adequacy for clinical research.

All original scale scores were standardized to generate comparable standardized scores = (factor per capita value/the full number of each item) × 100, with the exception of SF-36. This normalization procedure facilitated direct comparison across measurement instruments by converting all metrics to a common 0–100 scale.

### 2.4 Statistical analysis

Statistical analyses were performed using SPSS 26.0 and AMOS 21.0 software packages. Continuous variables (personality traits, 2-DSCS, SAS, SDS, and SF-36 scores) were confirmed normality by Kolmogorov–Smirnov test (all *p* > 0.05), and expressed as mean ± standard deviation (M ± SD), while categorical demographic variables (gender distribution, marital status, educational attainment, and healthcare payment capacity) were presented as frequencies and percentages. Bivariate correlation and linear regression analyses were conducted to assess multicollinearity. Results indicated that correlation coefficients among personality traits, emotional states, self-management behaviors, and quality of life dimensions were all < 0.8. Separate linear regression models were fitted with PCS and MCS as dependent variables and personality, emotional, and self-management dimensions as independent variables. All variance inflation factors (VIF) were <5, suggesting negligible multicollinearity.

A path analysis model was employed to construct the SEM for predicting the quality of life in individuals with T2DM, with an alpha level of 0.05 for entry into the model and 0.10 for exclusion from the model. Maximum likelihood estimation was utilized for parameter estimation, and mediation effects were tested using the Bootstrap method with 5,000 resamples. Statistical significance was indicated by a *p*-value of less than 0.05. The following indices were employed to evaluate the goodness-of-fit of hypothesized models: *χ*^2^/df < 5, Root Mean Square Error of Approximation (RMSEA < 0.08), Goodness-of-fit Index (GFI > 0.90), Adjusted Goodness-of-fit Index (AGFI > 0.90), Incremental fit Index (IFI > 0.90), Comparative fit Index (CFI > 0.90).

## 3 Results

The final analytical sample comprised 839 adults with T2DM recruited across 69 community health service centers, yielding an average of 12 participants per recruitment site (range: 8–15). All enrolled participants successfully completed the standardized assessment battery, with full compliance and complete data acquisition confirmed through rigorous quality control procedures.

### 3.1 Demographic characteristics of the participants

The study population (*N* = 839) had a mean age of 66.92 ± 8.67 years (M ± SD), with female participants (61.75%). The average diabetes duration was 8.87 ± 6.91 years, with 39.57% of participants reporting a disease duration ≤5 years. Educational attainment was relatively low, with 57.47% having completed primary school education or less. Financial constraints were reported 8.94% of participants, who indicated difficulty affording medical expenses (Table 1).

**Table 1:** Demographic characteristics of patients with T2DM.

Variables	Group	Mean ± SD/*N* (%)
Age		66.92 ± 8.67
Gender	Male	321(38.25)
	Female	518 (61.75)
Duration of hypertension (years)	≤5	332 (39.57)
	6–10	250 (29.80)
	11–15	124 (14.78)
	≥16	133 (15.85)
Marital status	Married	757 (90.22)
	Divorced	76 (9.06)
	widow	3 (0.35)
	Single	3 (0.35)
Education level	Primary School or below	457 (54.47)
	Middle school	249 (29.68)
	High school or higher	133 (15.85)
Healthcare payment capacity	Fully able to pay	285 (33.97)
	Basically no problem	479 (57.09)
	Relatively difficult	75 (8.94)

### 3.2 The scoring status of the big five personality traits, self-management behaviors, emotions, and QOL

The assessment of personality traits revealed a hierarchical pattern, with agreeableness emerging as the most prominent trait, followed sequentially by conscientiousness, extraversion, neuroticism, and openness. Analysis of self-management behaviors demonstrated significant variation across domains. Medication adherence represented the highest-performing domain, while regular exercise received the lowest-scored behavior. Intermediate scores were observed for: foot care, prevention and treatment of hyperglycemia and hypoglycemia, dietary self-management, blood glucose monitoring (Table 2). The sample stratified into three distinct self-management tiers: 68 patients (8.10%) displayed inadequate self-management, 212 patients (25.27%) exhibited moderate self-management, while the majority (*n* = 559, 66.63%) maintained effective self-management practices. Psychological assessment identified 171 cases (20.38%) meeting thresholds for anxiety, distributed across severity levels: mild (*n* = 135, 16.09%), moderate (*n* = 33, 3.93%), and severe (*n* = 3, 0.36%). Depression symptoms affected 240 patients (28.61%), with severity gradations of mild (*n* = 194, 23.12%), moderate (*n* = 41, 4.89%), and severe (*n* = 5, 0.60%).

**Table 2:** The situation of personality, self-management behavior, emotion and QOL.

Variables	Min	Max	Mean ± SD	Standardized score
Personality				
Agreeableness	3	18	13.58 ± 2.55	75.55
Conscientiousness	3	18	11.81 ± 2.70	64.48
Extraversion	3	18	11.62 ± 2.79	62.21
Neuroticism	3	18	6.78 ± 2.75	40.94
Openness	3	18	6.59 ± 2.65	38.73
Total score of 2-DSCS	26	130	88.73 ± 19.14	70.75
Dietary self-management	6	30	20.22 ± 5.79	49.83
Foot care	5	25	17.21 ± 4.54	54.12
Prevention and treatment of hyperglycemia and hypoglycemia	4	20	13.99 ± 4.22	51.86
Medication compliance	3	15	12.79 ± 2.76	67.33
Regular exercise	4	20	12.35 ± 4.89	39.66
Blood glucose monitoring	4	20	12.17 ± 4.10	45.98
SDS	20	74	36.36 ± 8.99	45.45
SAS	20	66	34.50 ± 7.51	43.13
SF-36				
SF	12.5	100	82.11 ± 16.71	–
PF	10	100	80.72 ± 16.91	–
RP	0	100	79.82 ± 35.05	–
RE	0	100	78.78 ± 36.92	–
BP	20	94	76.64 ± 16.61	–
MH	0	100	76.17 ± 17.25	–
VT	0	100	65.24 ± 19.26	–
GH	0	100	50.64 ± 20.69	–

### 3.3 The construction and testing of the structural equation model

Bivariate correlation analysis revealed significant associations among QOL dimensions, emotional states, and personality traits. Both psychological and physiological QOL dimensions were positively correlated with self-management behaviors, while being negatively correlated with anxiety symptoms, and depressive symptoms, neuroticism. Notably, the physiological QOL dimension showed a positive correlation with extroversion, whereas the psychological dimension was positively associated with conscientiousness and agreeableness. Emotional states exhibited consistent negative correlations with self-management behaviors (anxiety: *r* = −0.343; depression: *r* = −0.277) and agreeableness (*r* = −0.101 to −0.097), along with positive correlations with neuroticism. Depression was additionally negatively correlated with conscientiousness. Regarding behavioral correlations, self-management behaviors maintained negative associations with neuroticism and positive correlations with both conscientiousness and agreeableness.

**Figure 1:**
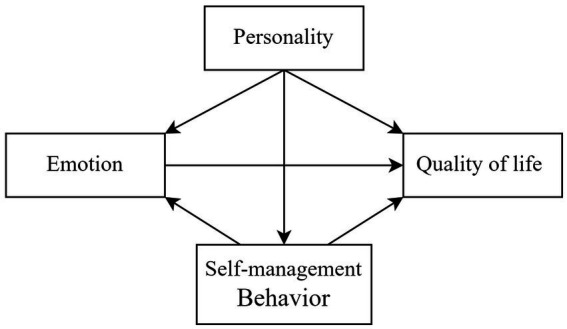
Initial hypothesis model of QOL among T2DM patents.

Building upon these empirical findings (Table 3), we propose a theoretically grounded conceptual model (Figure 1) that systematically integrates the interrelationships among personality traits, self-management behaviors, emotional states, and QOL domains in patients with T2DM. This integrative model not only elucidates the complex psycho-behavioral mechanisms underlying diabetes management but also offers an evidence-based framework for guiding both future research directions and clinical interventions targeting improved patient outcomes.

**Table 3:** Correlation among personality, self-management behavior, emotion and QOL.

Variables	PCS	MCS	2-DCCS	SAS	SDS
MCS	0.211**				
2-DCCS	0.222**	0.356**			
SAS	−0.342**	−0.507**	−0.343**		
SDS	−0.331**	−0.514**	−0.277**	0.732**	
Neuroticism	−0.122**	−0.119**	−0.073*	0.147**	0.181**
Conscientiousness	0.055	0.108**	0.109**	−0.054	−0.074*
Agreeableness	0.052	0.149**	0.189**	−0.101**	−0.097**
Openness	0.045	0.019	−0.023	−0.053	−0.005
Extraversion	0.100**	0.010	−0.065	−0.012	−0.065

*p < 0.05, **p < 0.01.

**Figure 2:**
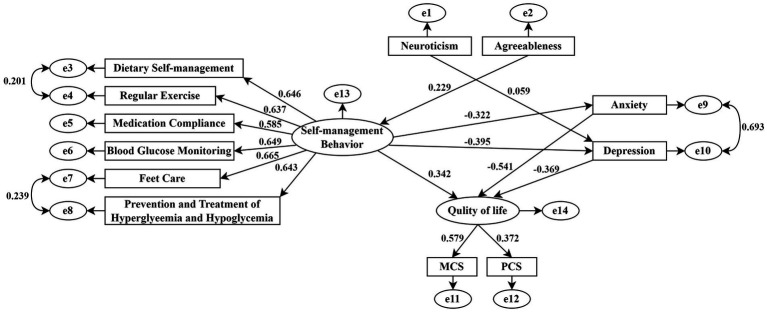
Structural equation model of QOL among T2DM Patients.

Parameter estimation was conducted using maximum likelihood estimation. Non-significant pathways (*p* > 0.05) were eliminated based on modification indices and standardized regression coefficients. Three categories of paths were removed: (1) all pathways connecting conscientiousness and extraversion to emotional states, self-management behaviors, and QOL; (2) associations between neuroticism and anxiety, self-management behaviors or QOL; and (3) relationships between agreeableness with emotional states and QOL. The optimized model demonstrated superior goodness of fit compared to the initial hypothesized model (Figure 2), with all fit indices (*χ*^2^/df = 3.556, RMSEA = 0.055, GFI = 0.967, AGFI = 0.946, IFI = 0.957, CFI = 0.957) meeting established criteria for model adequacy (Table 4).

**Table 4:** Goodness-of-fit indices of the structural equation model for QOL among T2DM patients.

Fit indices	*χ*^2^/df	RMSEA	GFI	AGFI	IFI	CFI
Structural equation model of QOL	3.556	0.055	0.967	0.946	0.957	0.957
Evaluation criterion	<5	<0.08	>0.90	>0.90	>0.90	>0.90

The modified model demonstrated statistically significant pathways (all |C. R.| > 1.96, *p* < 0.05), revealing several key associations. Neuroticism showed a significant positive association with depressive symptoms (*β* = 0.059, *p* < 0.05), while agreeableness was positively related to self-management behaviors (*β* = 0.229, *p* < 0.01). Self-management behaviors were inversely related to anxiety (β = −0.322, *p* < 0.01) and depression (*β* = −0.395, *p* < 0.01). Notably, standardized path coefficients indicated that anxiety had the strongest negative association with QOL (*β* = −0.541, *p* < 0.01), followed by depression (*β* = −0.369, *p* < 0.01), and self-management behaviors (*β* = 0.342, *p* < 0.01) (Table 5). These findings emphasize the pivotal role of psychological factors in QOL outcomes and suggest that comprehensive diabetes management should simultaneously address both emotional well-being and behavioral regulation.

**Table 5:** Path analysis results of QOL among T2DM patients.

Regression path	Standardized estimate	S. E.	C. R.	*P*
Depression ← Neuroticism	0.059	0.093	2.545	0.011
Self-management behavior ← Agreeableness	0.229	0.058	5.831	<0.01
Anxiety ← Self-management behavior	−0.322	0.101	−7.973	<0.01
Depression ← Self-management behavior	−0.395	0.122	−9.648	<0.01
QOL ← Anxiety	−0.541	0.024	−7.377	<0.01
QOL ← Depression	−0.369	0.019	−5.301	<0.01
QOL ← Self-management behavior	0.342	0.050	5.639	<0.01

### 3.4 The mediating effect of emotions between self-management behaviors and QOL

Structural equation modeling revealed significant mediation effects through both anxiety (*β* = 0.388, CI = 0.187, 0.320) and depression (*β* = 0.379, CI = 0.225, 0.378) in the relationship between self-management behaviors and QOL. These findings demonstrate that emotional states substantially mediate (accounting for 33.70% [anxiety] and 30.33% [depression] of total effects) the influence of diabetes self-management behaviors on QOL (Table 6). All mediation pathways were statistically significant (*p* < 0.01) based on bootstrap testing with 5,000 samples.

**Table 6:** The impact of emotions as a mediating variable on QOL.

Regression path	Standardized estimate	95%CI	S. E.	*P*
QOL ← Anxiety ← Self-management behavior	0.388	0.187 ~ 0.320	0.033	0.001
QOL ← Depression ← Self-management behavior	0.379	0.225 ~ 0.378	0.040	0.001

## 4 Discussion

This investigation employed structural equation modeling (SEM) to examine the complex interrelationships among personality, emotional states, self-management behaviors, and QOL in individuals with T2DM. As a prevalent chronic metabolic disorder affecting global populations, diabetes management necessitates robust self-regulation strategies encompassing dietary modifications, physical activity, and medication adherence, and all of which are critical for optimizing long-term health outcomes and patient well-being. Behavioral assessments revealed suboptimal adherence to exercise regimens and glycemic monitoring practices, with existing literature attributing these patterns to multiple factors including procedural discomfort, limited health literacy, and psychological barriers (Gao, 2018). Notably, patients prioritized pharmacological and dietary interventions, while comparatively undervaluing exercise therapy—an evidence-based but gradual-onset therapeutic modality requiring consistent long-term commitment (Wen et al., 2019).

### 4.1 Personality traits and emotional distress

The comprehensive assessment of T2DM patients identified agreeableness as the predominant personality dimension, reflecting characteristic psychosocial tendencies including trustworthiness, altruistic behaviors, and emotional empathy. This finding aligns with developmental psychology research demonstrating age-related increases in agreeableness (Li et al., 2020), potentially facilitating greater social engagement and interpersonal harmony in older adult populations. Path analysis further demonstrated that neuroticism exerted a substantial positive effect on depressive symptoms, indicating heightened to environmental stressors among high-neuroticism individuals. This finding aligns with well-established psychopathological models characterizing neuroticism as a vulnerability factor encompassing emotional instability, negative affectivity, and impaired stress coping mechanisms (Novak et al., 2017; van der Feltz-Cornelis et al., 2018).

### 4.2 Personality traits and self-management behaviors

Extensive evidence demonstrates that personality traits (stable patterns of cognition, emotion regulation, and behavior) (American Diabetes Association, 2018; van der Feltz-Cornelis et al., 2018) substantially shape lifestyle choices and health-related behaviors, particularly in exercise adherence and dietary patterns. The findings of this study revealed distinct associations: neuroticism correlated negatively with self-management behaviors and psychological dimensions of QOL, whereas conscientiousness and agreeableness showed positive correlations with both self-management behaviors and psychological dimensions of QOL. Path analysis provided additional support for the association between agreeableness and improved self-management behaviors. Highly agreeable individuals tend to demonstrate greater awareness of exercise benefits and higher dietary guideline compliance, which may be related to their characteristic altruism and cooperativeness (Li et al., 2020). These traits have also correlated with medication adherence (Abu et al., 2023). Moreover, these patients were more likely to exhibit stronger health responsibility and sustained commitment to long-term self-management goals. These findings underscore the importance of incorporating personality-adapted strategies in diabetes management, particularly implementing graduated behavioral objectives designed to systematically enhance patient self-efficacy. Clinically, patients with elevated neuroticism typically exhibit maladaptive behavioral patterns including impulsivity, delay discounting, and preference for immediate reinforcement. Existing evidence suggests that these behavioral manifestations may be mediated by stress-induced hormonal release, which selectively impairs prefrontal cortical functioning—a neural substrate critically involved in cognitive control, decision-making, and behavior regulation (Hirsh et al., 2008). Importantly, these neurocognitive disruptions appear to collectively compromise patients’ ability to maintain consistent adherence to essential diabetes self-management protocols, thereby adversely affecting long-term health outcomes (Deng et al., 2023).

### 4.3 The mediating role of emotional states

This study revealed anxiety (20.38%) and depression (28.61%) prevalence rates among T2DM patients, consistent with international epidemiological reports (e.g., 21.8% depression rate in Egyptian populations). The findings support the established bidirectional diabetes-mood disorder relationship, with elevated emotional problem rates in diabetics versus the general population (AlBekairy et al., 2017; Bragg et al., 2017; Briganti et al., 2018). Longitudinal evidence further indicates pre-existing mood disorders as independent risk factors for diabetes onset (Tabák et al., 2014). This study provides empirical evidence for the mediating role of emotional states in the relationship between self-management behaviors and QOL in patients with diabetes. This mediation may operate through several pathways: improved self-management competence enhances disease coping efficacy, potentially slowing disease progression and stabilizing clinical parameters, thereby reducing negative emotional states. Conversely, deficient self-management capacity often correlates with suboptimal clinical outcomes and elevated complication rates, which may precipitate emotional distress (Das et al., 2013). At the neuroendocrine level, anxiety and depression can dysregulate the hypothalamic–pituitary–target gland axis, promoting secretion of insulin-counterregulatory hormones and consequently impairing glycemic control. These physiological disturbances contribute substantially to the progression of diabetic complications and mortality risk, ultimately compromising QOL (Liu et al., 2020). Therefore, these findings underscore the necessity for comprehensive diabetes care that integrates: standard biomedical management, routine emotional health assessment, and evidence-based psychological interventions. Such multidimensional approaches may optimize disease trajectories and enhance overall patient wellbeing (Li et al., 2021).

The current findings highlight the crucial role of personality traits and emotional regulation in chronic disease management, suggesting that personalized psychological interventions customized to patients’ specific mental health profiles can significantly enhance disease self-management and improve QOL. Several evidence-based psychological interventions have demonstrated particular promise: Acceptance and Commitment Therapy (ACT) helps individuals with high neuroticism levels by cultivating acceptance of unavoidable negative emotions while promoting value-driven behavioral changes to alleviate diabetes-related distress (Bendig et al., 2022). Web-based Cognitive Behavioral Therapy (CBT) modules incorporating mindfulness techniques effectively support diabetes self-care behaviors while improving both glycemic control and depressive symptoms (Varela-Moreno et al., 2024). Mindfulness-based interventions, including Mindfulness-Based Cognitive Therapy (MBCT) and Mindfulness-Based Stress Reduction (MBSR), demonstrate efficacy in reducing anxiety, depressive symptoms, and diabetes-related stress in T2DM patients (Wang et al., 2025). For patients exhibiting impaired cardiac autonomic function, Heart Rate Variability Biofeedback (HRVB) serves as a valuable non-pharmacological approach to enhance autonomic nervous system activity while improving self-management behaviors and reducing depression (Wu et al., 2024). Furthermore, advancements in digital mental health interventions, such as AI-powered platforms like Woebot, provide comprehensive support through psychoeducation, mood monitoring, journaling features, and real-time interactive counseling for emotional regulation and stress reduction (Hoffman et al., 2023).

### 4.4 Limitations and future directions

Two primary limitations warrant consideration in interpreting these findings. First, the exclusive recruitment of participants from a structured self-management intervention program suggests potential selection bias toward individuals with pre-existing behavioral motivation and established self-care capacity, thereby potentially limiting the results for less motivated patient subgroups within the broader T2DM population. Second, the observational, cross-sectional design precludes definitive causal inferences regarding the relationship among examined variables, necessitating future experimental or longitudinal investigations to elucidate underlying causal mechanisms and directional relationships.

## 5 Conclusion

This investigation systematically delineates the complex network of interrelationships connecting personality traits, emotional states, self-management behaviors, and QOL in patients with T2DM. The study substantiates the crucial mediating function of emotional regulation in linking self-management behaviors and QOL, thereby underscoring the clinical imperative of integrating psychological support into standard diabetes care protocols. These evidence-based findings offer strategies to optimize wellbeing in T2DM patients. While providing valuable insights, the current research design—characterized by selective sampling and cross-sectional methodology-necessitates future prospective studies to verify the temporal dynamics and causal pathways underlying these observed relationships.

## Data Availability

The authors of this article were authorized to conduct statistical analysis of the data but held no ownership rights. According to the relevant local data management regulations, data sharing required approval from the local health administrative department and the Hangzhou Center for Disease Control and Prevention (Hangzhou Health Supervision Institute). Requests to access the datasets should be directed to Wen Fu, fwseven20@aliyun.com.

## References

[ref1] Abu E. K. Antiri E. O. Ocansey S. Ntodie M. Abokyi S. Abraham C. H. (2023). Associations between personality traits and adherence to treatment in patients with primary open-angle glaucoma in an African population. Clin. Exp. Optom. 106, 509–515. doi: 10.1080/08164622.2022.2075253, PMID: 35645224

[ref2] AlBekairy A. AbuRuz S. Alsabani B. Alshehri A. Aldebasi T. Alkatheri A.. (2017). Exploring factors associated with depression and anxiety among hospitalized patients with type 2 diabetes mellitus. Med. Princ. Pract. 26, 547–553. doi: 10.1159/000484929, PMID: 29131123 PMC5848470

[ref3] American Diabetes Association (2018). Lifestyle management: standards of medical care in diabetes-2018. Diabetes Care 41, S38–S50. doi: 10.2337/dc18-S004 29222375

[ref4] Babazadeh T. Dianatinasab M. Daemi A. Nikbakht H. A. Moradi F. Ghaffari-Fam S. (2017). Association of self-care behaviors and quality of life among patients with type 2 diabetes mellitus: Chaldoran County, Iran. Diabetes Metab. J. 41, 449–456. doi: 10.4093/dmj.2017.41.6.449, PMID: 29272083 PMC5741554

[ref5] Basiri R. Seidu B. Rudich M. (2023). Exploring the interrelationships between diabetes, nutrition, anxiety, and depression: implications for treatment and prevention strategies. Nutrients 15:4226. doi: 10.3390/nu15194226, PMID: 37836510 PMC10574484

[ref6] Bayani M. A. Shakiba N. Bijani A. Moudi S. (2022). Depression and quality of life in patients with type 2 diabetes mellitus. Caspian J. Intern. Med. 13, 335–342. doi: 10.22088/cjim.13.2.3, PMID: 35919653 PMC9301220

[ref7] Bendig E. Schmitt A. Wittenberg A. Kulzer B. Hermanns N. Moshagen M.. (2022). ACTonDiabetes: study protocol of a pragmatic randomised controlled trial for the evaluation of an acceptance and commitment-based internet-based and mobile-based intervention for adults living with type 1 or type 2 diabetes. BMJ Open 12:e059336. doi: 10.1136/bmjopen-2021-059336, PMID: 36109030 PMC9478835

[ref8] Bragg F. Holmes M. V. Iona A. Guo Y. Du H. Chen Y.. (2017). Association between diabetes and cause-specific mortality in rural and urban areas of China. JAMA 317, 280–289. doi: 10.1001/jama.2016.19720, PMID: 28114552 PMC6520233

[ref9] Briganti C. P. Silva M. T. Almeida J. V. Bergamaschi C. C. (2018). Association between diabetes mellitus and depressive symptoms in the Brazilian population. Rev. Saude Publica 53:5. doi: 10.11606/s1518-8787.2019053000608, PMID: 30652778 PMC6391867

[ref10] Chinese Diabetes Society (2021). Guideline for the prevention and treatment of type 2 diabetes mellitus in China (2020 edition). Chin. J. Diabetes Mellitus 13, 315–409. doi: 10.3760/cma.j.cn115791-20210221-00095

[ref11] Conti C. Di Francesco G. Fontanella L. Carrozzino D. Patierno C. Vitacolonna E.. (2017). Negative affectivity predicts lower quality of life and metabolic control in type 2 diabetes patients: a structural equation modeling approach. Front. Psychol. 8:831. doi: 10.3389/fpsyg.2017.00831, PMID: 28596745 PMC5443140

[ref1002] Deng L. Luo S. Fang Q. Xu J. (2023). Intertemporal decision-making as a mediator between personality traits and self-management in type 2 diabetes: a cross-sectional study. Front. Psychol. 28, 1210691. doi: 10.3389/fpsyg.2023.1210691, PMID: 37575446 PMC10422026

[ref12] Dadras Z. Molaei B. Aghamohammadi M. (2022). The relationship between personality profile and self-care among patients with type 2 diabetes. Front. Psychol. Nov 15, 13:1030911. doi: 10.3389/fpsyg.2022.1030911, PMID: 36457923 PMC9706217

[ref13] Das R. Singh O. Thakurta R. G. Khandakar M. R. Ali S. N. Mallick A. K.. (2013). Prevalence of depression in patients with type II diabetes mellitus and its impact on quality of life. Indian J. Psychol. Med. 35, 284–289. doi: 10.4103/0253-7176.119502, PMID: 24249932 PMC3821207

[ref14] De Groot M. Crick K. A. Long M. Saha C. Shubrook J. H. (2016). Lifetime duration of depressive disorders in patients with type 2 diabetes. Diabetes Care 39, 2174–2181. doi: 10.2337/dc16-1145, PMID: 27729427 PMC5127229

[ref15] Gaffari-Fam S. Lotfi Y. Daemi A. Babazadeh T. Sarbazi E. Dargahi-Abbasabad G.. (2020). Impact of health literacy and self-care behaviors on health-related quality of life in Iranians with type 2 diabetes: a cross-sectional study. Health Qual. Life Outcomes 18:357. doi: 10.1186/s12955-020-01613-8, PMID: 33148266 PMC7640476

[ref16] Gao S. S. (2018). Analysis of self-management behaviors and influencing factors in elderly patients with type 2 diabetes. Chin. Genl. Pract. Nurs. 16, 816–818. doi: 10.3969/j.issn.1674-4748.2018.07.020

[ref17] Hall P. A. Rodin G. M. Vallis T. M. Perkins B. A. (2009). The consequences of anxious temperament for disease detection, self-management behavior, and quality of life in type 2 diabetes mellitus. J. Psychosom. Res. 67, 297–305. doi: 10.1016/j.jpsychores.2009.05.015, PMID: 19773022

[ref18] Hirsh J. B. Morisano D. Peterson J. B. (2008). Delay discounting: interactions between personality and cognitive ability. J. Res. Pers. 42, 1646–1650. doi: 10.1016/j.jrp.2008.07.005

[ref19] Hoffman V. Flom M. Mariano T. Y. Chiauzzi E. Williams A. Kirvin-Quamme A.. (2023). User engagement clusters of an 8-week digital mental health intervention guided by a relational agent (Woebot): exploratory study. J. Med. Internet Res. 25:e47198. doi: 10.2196/47198, PMID: 37831490 PMC10612009

[ref20] Hughes D. J. Kratsiotis I. K. Niven K. Holman D. (2020). Personality traits and emotion regulation: a targeted review and recommendations. Emotion 20, 63–67. doi: 10.1037/emo0000644, PMID: 31961180

[ref21] Lang X. Huang S. Xiao Y. (2025). The relationship between personality and self-management behavior in Chinese young and middle-aged people with chronic illness: the chain mediating role of family health and health literacy. Patient Prefer. Adherence 19, 997–1009. doi: 10.2147/PPA.S507666, PMID: 40235830 PMC11998933

[ref22] Li Z. M. Gao M. Chen X. Y. Sun X. Y. (2020). Relationship between the five-factor model of personality traits and self-management attitude of patients with type 2 diabetes. Beijing Da Xue Xue Bao 52, 506–513. doi: 10.19723/j.issn.1671-167X.2020.03.017, PMID: 32541985 PMC7433411

[ref23] Li M. F. Li X.-y. Liang B. (2021). Anxiety and depression in patients with type 2 diabetes mellitus and their influencing factors. Chin. J. Gen. Pract. 19, 1135–1146. doi: 10.16766/j.cnki.issn.1674-4152.002004

[ref24] Liu X. Haagsma J. Sijbrands E. Buijks H. Boogaard L. Mackenbach J. P.. (2020). Anxiety and depression in diabetes care: longitudinal associations with health-related quality of life. Sci. Rep. 10:8307. doi: 10.1038/s41598-020-57647-x, PMID: 32433470 PMC7239869

[ref25] National Health Commission Disease Control and Prevention Bureau (2021). Report on nutrition and chronic diseases of Chinese residents (2020). Beijing: People’s Publishing House.

[ref26] Novak J. R. Anderson J. R. Johnson M. D. Hardy N. R. Walker A. Wilcox A.. (2017). Does personality matter in diabetes adherence? Exploring the pathways between neuroticism and patient adherence in couples with type 2 diabetes. Appl. Psychol. Health Well Being 9, 207–227. doi: 10.1111/aphw.12087, PMID: 28401663 PMC5511078

[ref27] Ory M. G. Han G. Nsobundu C. Carpenter K. Towne S. D. Jr. Smith M. L. (2025). Comparative effectiveness of diabetes self-management education and support intervention strategies among adults with type 2 diabetes in Texas. Front. Public Health 13:1543298. doi: 10.3389/fpubh.2025.1543298, PMID: 40171438 PMC11959030

[ref28] Ozdemir N. Sahin A. Z. (2020). Anxiety levels, quality of life and related socio-demographic factors in patients with type 2 diabetes. Niger. J. Clin. Pract. 23, 775–782. doi: 10.4103/njcp.njcp_523_19, PMID: 32525111

[ref29] Salzwedel A. Koran I. Langheim E. Schlitt A. Nothroff J. Bongarth C.. (2020). Patient-reported outcomes predict return to work and health-related quality of life six months after cardiac rehabilitation: results from a German multi-Centre registry (OutCaRe). PLoS One 15:e0232752. doi: 10.1371/journal.pone.0232752, PMID: 32369514 PMC7199966

[ref30] Schindler S. Querengässer J. (2019). Coping with sadness: how personality and emotion regulation strategies differentially predict the experience of induced emotions. Pers. Individ. Differ. 136, 90–95. doi: 10.1016/j.paid.2018.01.050

[ref31] Speight J. Holmes-Truscott E. Hendrieckx C. Skovlund S. Cooke D. (2020). Assessing the impact of diabetes on quality of life: what have the past 25 years taught us? Diabet. Med. 37, 483–492. doi: 10.1111/dme.14196, PMID: 31797443

[ref32] Stevanovic D. Habtewold T. D. Niksi A. Avicena M. Knez R. (2019). Anxiety and depressive disorders in diabetes: Sadikot’s International Textbook of Diabetes.

[ref33] Sun H. Saeedi P. Karuranga S. Pinkepank M. Ogurtsova K. Duncan B. B.. (2022). IDF diabetes atlas: global, regional and country-level diabetes prevalence estimates for 2021 and projections for 2045. Diabetes Res. Clin. Pract. 183:9119. doi: 10.1016/j.diabres.2021.109119, PMID: 34879977 PMC11057359

[ref34] Tabák A. G. Akbaraly T. N. Batty G. D. Kivimäki M. (2014). Depression and type 2 diabetes: a causal association? Lancet Diabetes Endocrinol. 2, 236–245. doi: 10.1016/s2213-8587(13)70139-6, PMID: 24622754

[ref35] Tan H. Q. M. Chin Y. H. Ng C. H. Liow Y. Devi M. K. Khoo C. M.. (2020). Multidisciplinary team approach to diabetes. An outlook on providers’ and patients’ perspectives. Prim. Care Diabetes 14, 545–551. doi: 10.1016/j.pcd.2020.05.012, PMID: 32591227

[ref36] Teli M. Thato R. Rias Y. A. (2023). Predicting factors of health-related quality of life among adults with type 2 diabetes: a systematic review. SAGE Open Nurs. 9:23779608231185921. doi: 10.1177/23779608231185921, PMID: 37448972 PMC10336768

[ref37] van der Feltz-Cornelis C. M. Simmons A. E. N. de Jonge P. (2018). Chapter 5-personality and type 2 diabetes: an overview of the epidemiological evidence. New York: Personality and Disease.

[ref38] Varela-Moreno E. Anarte-Ortiz M. T. Jodar-Sanchez F. Garcia-Palacios A. Monreal-Bartolome A. Gili M.. (2024). Economic evaluation of a web application implemented in primary Care for the Treatment of depression in patients with type 2 diabetes mellitus: multicenter randomized controlled trial. JMIR Mhealth Uhealth 12:e55483. doi: 10.2196/55483, PMID: 38754101 PMC11140277

[ref39] Wang H. Ge L. Kwok Y. Y. Zhang Z. Wiley J. Guo J. (2025). A blended mindfulness-based stress reduction program to improve diabetes self-management among people with type 2 diabetes mellitus: a mediation effect analysis. Ann. Behav. Med. 59:kaae075. doi: 10.1093/abm/kaae075, PMID: 39657759

[ref40] Ware J. E. Kosinski M. A. Keller S. D. (1996). A 12-item short-form health survey: construction of scales and preliminary tests of reliability and validity. Med. Care 34, 220–233. doi: 10.1097/00005650-199603000-00003, PMID: 8628042

[ref41] Ware J. E. Sherbourne C. D. (1992). The MOS 36-item short-form health survey (SF-36). I. Conceptual framework and item selection. Med. Care 30, 473–483. 1593914

[ref42] Wen Z. L. Jiang K. W. Jiang X. (2019). Comprehensive evaluation and self-management of diabetes care for the elderly. Chin. J. Health Manage. 13, 165–169. doi: 10.3760/cma.j.issn.1674-0815.2019.02.016

[ref43] Wolf E. J. Harrington K. M. Clark S. L. Miller M. W. (2013). Sample size requirements for structural equation models: an evaluation of power, Bias, and solution propriety. Educ. Psychol. Meas. 76, 913–934. doi: 10.1177/0013164413495237, PMID: 25705052 PMC4334479

[ref44] Wu Y. R. Su W. S. Lin K. D. Lin I. M. (2024). Effect of heart rate variability biofeedback on cardiac autonomic activation and diabetes self-care in patients with type II diabetes mellitus. Appl. Psychophysiol. Biofeedback 50, 315–327. doi: 10.1007/s10484-024-09666-x 39342048

[ref45] Yan M. Yuan L. (2016). Investigation and analysis of depression and anxiety in patients with diabetes mellitus. Chin. J. Mod. Nurs. 22, 1086–1089. doi: 10.3760/cma.j.issn.1674-2907.2016.08.012

[ref46] Zhang X. T. Wang M. C. He L. N. Jie L. Deng J. X. (2019). The development and psychometric evaluation of the Chinese big five personality Inventory-15. PLoS One 14:e0221621. doi: 10.1371/journal.pone.0221621, PMID: 31454383 PMC6771307

[ref47] Zung W. W. (1965). A self-rating depression scale. Arch. Gen. Psychiatry 12, 63–70. doi: 10.1001/archpsyc.1965.01720310065008, PMID: 14221692

[ref48] Zung W. W. (1971). A rating instrument for anxiety disorders. Psychosomatics 12, 371–379. doi: 10.1016/s0033-3182(71)71479-0, PMID: 5172928

